# Interventions Adopting Body-Mind-Spirit Model to Improve the Holistic Health of Older Adults: A Systematic Review

**DOI:** 10.1093/geroni/igab046.3409

**Published:** 2021-12-17

**Authors:** Xinyue Hu, Tongtong Li, Iris Chi

**Affiliations:** 1 University of California, Los Angeles, Los Angeles, California, United States; 2 University of Southern California, Arcadia, California, United States; 3 University of Southern California, University of Southern California, California, United States

## Abstract

This systematic review aims to summarize 5 key information from non-pharmaceutical intervention studies which adopt Body-Mind-Spirit (BMS) model for older adults: (1) definition of BMS, (2) types and formats of the interventions, (3) background and BMS training of the interventionists, (4) activities included in the interventions, and (5) effect of these interventions on the holistic health of older adults. We conducted a systematic search of 9 databases (ProQuest, Web of Science, PsycINFO, PubMed, Cochrane, Wanfang, AIRITI, CADAL, CNKI) for studies published in English or Chinese through May 31, 2021. Inclusion criteria were: (1) Must be empirical studies; (2) Participants must be aged 55 and above; and (3) Must adopt the BMS model or contain BMS in full-text. We found 15 studies (7 RCTs, 1 cluster randomized trial, 3 mixed-method studies, and 4 qualitative studies). Ten studies (66.67%) adopted Chan’s BMS model. Thirteen studies (86.67%) adopted in-person group interventions. Only five studies (33.33%) provided BMS training to the interventionists. Six articles (40%) categorized the activities as body-, mind- or spirituality-related. Ten studies (66.67%) reported effectiveness in all 3 dimensions of BMS. Of the 7 RCTs, 5 were rated as medium-quality, and 2 were rated as low-quality according to the Cochrane’s Risk of Bias tool. Most interventions based on the BMS model claimed to be effective in improving the holistic health of older adults. In order to improve the internal validity, future RCT studies should be more prudent about the randomization process and adhere to the BMS model when designing the interventions.

